# Profiling of circulating microRNAs in patients with Barrett’s esophagus and esophageal adenocarcinoma

**DOI:** 10.1007/s00535-015-1133-5

**Published:** 2015-11-19

**Authors:** Pauline Bus, Christine Kestens, Fiebo Jan Willem Ten Kate, Wilbert Peters, Joost Paulus Hubertus Drenth, Jeanine Merel Leonoor Roodhart, Peter Derk Siersema, Jantine Wilhelmina Paula Maria van Baal

**Affiliations:** Department of Gastroenterology and Hepatology, University Medical Center Utrecht, Heidelberglaan 100, 3584 CX Utrecht, The Netherlands; Department of Pathology, University Medical Center Utrecht, Heidelberglaan 100, 3584 CX Utrecht, The Netherlands; Department of Gastroenterology and Hepatology, Radboud University Medical Center, Geert Grooteplein 8, 6525 GA Nijmegen, The Netherlands; Department of Medical Oncology, University Medical Center Utrecht, Heidelberglaan 100, 3584 CX Utrecht, The Netherlands

**Keywords:** Circulating biomarker, MicroRNA, Plasma, Barrett’s esophagus, Esophageal adenocarcinoma

## Abstract

**Background:**

Circulating microRNAs (miRNAs) have been suggested as novel markers for various diseases. The goal of this pilot study was to identify circulating miRNAs differentially expressed comparing Barrett’s esophagus (BE), esophageal adenocarcinoma (EAC), and controls.

**Methods:**

MicroRNA expression profiling was performed by qPCR array using plasma from six controls and eight BE and eight EAC patients. Validation was performed by analyzing the expression of six selected miRNAs, by qRT-PCR in 115 plasma samples of controls, BE, and EAC patients. Diagnostic accuracy was evaluated by area under the curve (AUC) analysis.

**Results:**

We identified three miRNAs that were elevated in EAC and four miRNAs that were elevated in BE. Further validation showed that miRNA-382-5p was significantly increased and miRNA-133a-3p significantly decreased in EAC. miRNA-194-5p and miRNA-451a were significantly increased and miRNA-136-5p significantly decreased in BE versus controls. A combination of three or more miRNAs was found to have a good diagnostic performance in discriminating BE from controls (AUC: 0.832), EAC from controls (AUC: 0.846), and BE from EAC (AUC: 0.797).

**Conclusion:**

Our data suggest that circulating miRNAs are differentially expressed in BE and EAC. The miRNAs identified may be used for future non-invasive screening of BE and EAC.

**Electronic supplementary material:**

The online version of this article (doi:10.1007/s00535-015-1133-5) contains supplementary material, which is available to authorized users.

## Introduction

Barrett’s esophagus (BE) is a premalignant condition of the distal esophagus, characterized by the metaplastic replacement of normal stratified squamous epithelium by specialized intestinal columnar epithelium [1]. BE is associated with an increased risk of progression to esophageal adenocarcinoma (EAC) with an estimated annual incidence of 0.33 % [2]. During the last few decades, incidence rates of BE and EAC have increased dramatically in the Western world [3].

The current approach of BE screening includes routine surveillance endoscopy with histopathological analysis of biopsies taken during endoscopy. For the diagnosis of BE and EAC, morphological assessment of biopsies is the golden standard. The histological presence or absence of dysplasia is the basis for the advised endoscopic follow-up intervals in BE. Unfortunately, sampling bias occurs [4], and there is an ongoing discussion on whether BE surveillance is indeed cost-effective [5]. Additionally, histological grading of BE biopsies is subject to considerable interobserver variability [6, 7]. Moreover, in over 90 % of new EAC cases, BE was not diagnosed before [8]. This suggests that it is important to have easy-to-use markers that are able to distinguish BE and EAC patients from the normal population.

MicroRNAs (miRNAs) are small non-coding RNAs of approximately 21–25 nucleotides long. They function by negatively influencing the protein translation machinery via translational repression or mRNA degradation [9]. Expression profiles of miRNAs in various cancers have been investigated, showing that miRNA expression profiles are disease- and tissue-specific and, therefore, can be considered candidate biomarkers [10–13]. In addition, miRNA expression profiling of BE and EAC biopsies has shown tissue-specific miRNAs [14–17].

The stability and predictive value of miRNAs make them ideal to be tested in plasma or serum samples [18, 19]. Unique circulating miRNA profiles have been identified in patients with colorectal, breast, and esophageal squamous cell cancer (ESCC) [20–25]. However, the expression of circulating miRNAs in BE and EAC in plasma has as yet not been evaluated. We hypothesized that different miRNA expression patterns are present in plasma of BE and EAC patients compared to controls. We, therefore, determined circulating miRNA expression profiles in BE and EAC patients and in controls. Based on this, we selected a panel of miRNAs that was subsequently validated in a different and larger group of BE and EAC patients.

## Materials and methods

### Ethics statement

This study was approved by the medical ethical committee of the University Medical Center Utrecht and Radboud University Medical Center. Written consent was obtained from all patients prior to obtaining plasma samples.

### Study subjects

All participants, including controls, underwent routine endoscopy. Twelve milliliters of blood for plasma miRNA analysis was collected in EDTA-containing tubes (BD Biosciences, Plymouth, UK). Plasma from EAC patients was collected before treatment. All BE patients were on long-term proton pump inhibitors (PPIs) in a dose of 40–80 mg daily to treat and/or prevent reflux esophagitis. Controls were patients undergoing endoscopy for unexplained upper abdominal complaints, had no reflux symptoms or endoscopic abnormalities, and did not use PPIs. Biopsies were taken from controls and BE and EAC patients for histopathological confirmation. Histopathology confirmed that all BE patients had proven BE (with intestinal metaplasia) without dysplasia, controls had no signs of inflammation, and EAC patients had confirmed BE and EAC. Patient characteristics are summarized in Tables 1 and 2 and the study design in Suppl. Fig. 1.

**Table 1:** Characteristics of normal controls and patients with Barrett’s esophagus or esophageal adenocarcinoma used for microRNA expression profiling

Variable	Controls (*n* = 6)	BE (*n* = 8)	EAC (*n* = 8)
Gender (male)	3 (50 %)	7 (88 %)	7 (88 %)
Age (years, mean ± SD and range)	51.8 ± 10.3 (40–68)	59.5 ± 12.1 (40–76)	56.0 ± 11.0 (42–70)
Barrett length (mean and range)	na	C4M5 (C1M3–C10M11)	C8M8 (C5M5–C13M13)
Tumor stage (*T*)			
1	na	na	0 (0 %)
2			2 (25 %)
3			5 (63 %)
4			1 (13 %)
*X* (unknown)			0 (0 %)
Lymph node status (*N*)			
0	na	na	3 (30 %)
1			2 (25 %)
2			0 (0 %)
3			3 (38 %)
4			0 (0 %)
*X* (unknown)			0 (0 %)
Metastasis (*M*)			
0	na	na	1 (13 %)
1			1 (13 %)
*X* (unknown)			6 (75 %)
Body mass index (mean ± SD)	26.8 ± 5.3	25.2 ± 2.1	24.1 ± 1.9
Current smokers	1	2	1
Previous smokers	1	2	4
Non-smokers	4	4	3

*BE* Barrett’s esophagus, *EAC* esophageal adenocarcinoma, *CM* circumference length, maximum length

**Table 2:** Characteristics of normal controls and patients with Barrett’s esophagus or esophageal adenocarcinoma used for validation by Q-RT-PCR

Variable	Controls (*n* = 15)	BE (*n* = 41)	EAC (*n* = 59)
Gender (male)	3 (20 %)	31 (76 %)	53 (90 %)
Age (years, mean ± SD and range)	39.5 ± 14.7 (19–68)	59.2 ± 11.7 (35–81)	65.8 ± 10.8 (42–85)
Barrett length (mean and range)	na	C3M4 (C0M2–C10M11)	C5M8 (C0M2–C14M14)
Tumor stage (*T*)			
1	na	na	6 (10 %)
2			10 (17 %)
3			36 (61 %)
4			1 (2 %)
*X* (unknown)			6 (10 %)
Lymph node status (*N*)			
0	na	na	22 (37 %)
1			24 (41 %)
2			6 (10 %)
3			1 (2 %)
4			0 (0 %)
*X*			6 (10 %)
Metastasis (*M*)			
0	na	na	6 (10 %)
1			8 (14 %)
*X*			45 (76 %)
Body mass index (mean ± SD)	24.5 ± 4.0	25.6 ± 2.6	25.3 ± 2.9
Current smokers	3	6	4
Previous smokers	3	4	7
Non-smokers	9	6	6
Unknown		25	42

*BE* Barrett’s esophagus, *EAC* esophageal adenocarcinoma, *CM* circumference length, maximum length

Blood samples were centrifuged for 10 min at 2100*g* at 4 °C and plasma was collected. Samples were stored at −80 or −20 °C, before subsequent miRNA expression analysis.

### RNA isolation

RNA was isolated as previously described [26, 27] and according to the manufacturer’s protocol using the miRNeasy Mini kit (Qiagen, Venlo, the Netherlands).

Samples were defrosted on ice and centrifuged at 3000*g* for 5 min to remove residual platelets. Two hundred microliters of plasma was transferred into a new tube, and 3.75-volume Qiazol (Qiagen, Venlo, the Netherlands) containing 1.25 μg/mL MS2 RNA (Roche, Mannheim, Germany) was added. After 5 min incubation at room temperature, 0.2-volume chloroform (Merck, Darmstadt, Germany) was added. After centrifugation at 12,000*g* for 15 min at 4 °C, supernatant was transferred to a clean tube, and 1.5-volume 100 % ethanol (Merck) was added. The sample was then applied directly to a Qiagen RNeasy Mini Spin Column. The isolated RNA was dissolved in 30 μL RNase-free water.

### Quality control and miRNA expression profiling

RNA quality control and subsequent miRNA expression profiling were performed by Exiqon, Denmark. For quality control, 2 µL RNA was reverse transcribed (RT) in 10 µL reactions using the miRCURY LNA™ Universal RT microRNA polymerase chain reaction (PCR), Polyadenylation and cDNA synthesis kit (all from Exiqon). Each reverse transcription reaction was performed in duplicate, including an artificial RNA spike-in (Sp6, Exiqon). cDNA was diluted 50× and assayed in 10 µL PCR reactions according to the protocol for miRCURY LNA™ Universal RT microRNA PCR; 4 miRNAs (miRNA-103a-3p, miRNA-191-5p, miRNA-423-3p, and miRNA-451a) and Sp6 were assayed by quantitative polymerase chain reaction (qPCR). The amplification was performed in a Lightcycler^®^ 480 Real-Time PCR System (Roche). The amplification curves were analyzed using the Roche LC software, both for determination of Cp (∆∆Cp method) and for melting curve analysis. A mean Cp was calculated for the duplicate RTs and evaluation of expression levels was performed based on raw Cp values. High technical quality was achieved, since all four miRNAs and the synthetic spike-in were found to be present in the samples.

For miRNA expression profiling, 5 µL RNA was reverse transcribed in 25 µL reactions using the miRCURY LNA™ Universal RT microRNA PCR, Polyadenylation, and cDNA synthesis kit (Exiqon). cDNA was diluted 50× and assayed in 10 µL PCR reactions. PCR panels containing primers for miRNAs found in serum and plasma were used (Serum/Plasma Focus miRNA PCR panels, Exiqon). This panel consisted of 175 miRNAs that are known to be present in human plasma samples. Negative controls, samples excluding template in the RT reaction, were included.

### Data analysis, miRNA expression profiling

The amplification efficiency was calculated using algorithms similar to the LinReg software [28]. All assays were inspected for distinct melting curves. miRNA assays were included if the samples were detected five Cps lower than the negative control, the upper limit of detection was set to Cp 37. NormFinder was used to find the best normalizer [29]. Based on this, data were normalized to the average of assays detected in all samples [30]. Statistical analysis was performed using Kruskal–Wallis and Mann–Whitney *U* tests, depending on the number of groups tested. Fold changes were measured using mean ratios. miRNAs with a *p* value of 0.05 or lower or fold changes of 1.5 or higher were listed and supposed to be differentially expressed between the various groups.

### Validation by real-time reverse transcribed polymerase chain reaction

Six miRNAs were selected from the initial miRNA profiling phase for further validation by real-time reverse transcribed polymerase chain reaction (RT-PCR) assays. Selection criteria are described in Suppl. Fig. 2. In addition, NormFinder was used on the initial circulating miRNA profiling results to select a set of miRNAs that could be used for normalization [30]. miRNA-93-5p, -103a-3p, -106a-5p, -423-3p, and -423-5p were used for normalization (Suppl Fig. 3).

RT-PCR assays were performed by three experienced researchers. The analyses were performed by using the TaqMan MicroRNA Assay kit according to the manufacturer’s protocol (Applied Biosystems, CA, USA). All RT and PCR reactions were performed on a thermal cycler (iCycler iQ system, BioRad, Hercules, CA, USA). Assays were performed in duplicate (technical replicates).

### Data analysis, validation study

The relative expression level was calculated using the ∆Ct method. miRNA expression was expressed in box plots. Data was analyzed using GraphPad Prism version 5.03 (San Diego, CA, USA). The groups were analyzed using Kruskal–Wallis and Mann–Whitney *U* tests, depending on the number of groups compared. Fold changes were measured using mean ratios. To evaluate whether circulating miRNAs could be used to distinguish between the different groups receiver operating characteristic (ROC) curve analysis was performed to determine the accuracy of the established miRNA panel [area under the curve (AUC), 95 % confidence interval (CI), sensitivity, and specificity] (GraphPad Prism). Logistic regression analysis (Statistical Package for Social Sciences, version 20; SPSS Inc., Chicago, IL, USA) was used to determine miRNA panels for ROC curve analysis.

## Results

### miRNA expression profiling

Three unique circulating miRNA profiles of plasma samples from BE and EAC patients and controls were obtained. On average, 161 miRNAs were detected in each sample, with an overlap of 114 miRNAs in all samples. The complete circulating miRNA expression profiles can be found on the Gene Expression Omnibus website (http://www.ncbi.nlm.nih.gov/geo/, accession code: GSE51410).

**Fig. 1:**
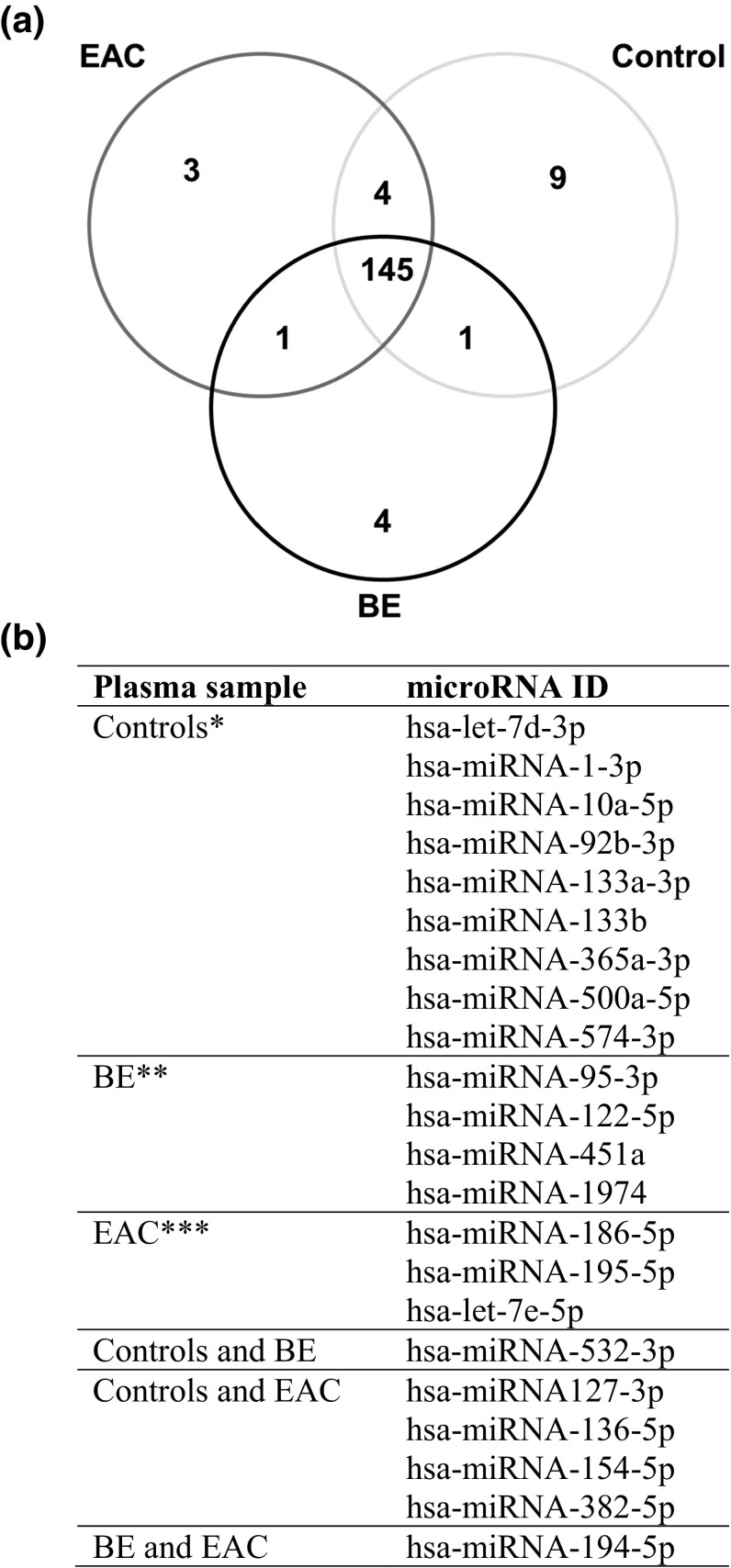
Venn diagram of circulating microRNAs in esophageal adenocarcinoma, Barrett’s esophagus, and normal controls; profiling study. Venn diagram showing the distribution of microRNA expression comparing plasma samples from controls, patients with *BE* Barrett’s esophagus, and *EAC* esophageal adenocarcinoma. **a** Depicted are all miRNAs significantly differentially expressed or more than 1.5-fold up- or down-regulated comparing the various groups. **b** Shows the specific microRNAs depicted in the Venn diagram

A Venn diagram (Fig. 1a) was constructed to sort the distribution of the miRNAs in plasma samples from BE and EAC patients and controls. The diagram demonstrated that eight miRNAs were elevated in plasma samples from controls, four miRNAs were elevated in plasma samples from BE, and three miRNAs were elevated in plasma samples from EAC. The specific miRNAs presented in the Venn diagram are shown in Fig. 1b. Comparing the various groups, we found that miRNA-194-5p was highly present in plasma samples from EAC and BE patients compared to controls. miRNA-532-3p was highly expressed in both BE and controls compared to EAC, while miRNA-136-5p, -127-3p, -154-5p, and -382-5p were highly present in EAC and controls compared to BE (Fig. 1b).

### miRNAs differentially expressed in plasma from BE and EAC patients and controls

We first investigated the miRNAs that were differentially expressed when comparing plasma samples from BE with controls (Table 3). Five miRNAs were more than 1.5-fold up-regulated, and eight miRNAs were more than 1.5-fold down-regulated comparing BE with controls. In addition, two miRNAs were significantly differentially expressed, of which one miRNA was up-regulated and one miRNA was down-regulated in BE compared to controls (miRNA-451a and let-7d-3p, respectively).

**Table 3:** MicroRNA expression in plasma samples; profiling study

MicroRNA ID	Mean expression controls	Mean expression BE	Mean expression EAC	Fold induction BE/controls	Fold induction EAC/controls	Fold induction EAC/BE
hsa-let-7d-3p	1.02	0.78	0.85	0.76 (*p* = 0.011)	0.83	1.09
hsa-let-7e-5p	1.22	1.08	1.61	0.88	1.32	1.50 (*p* = 0.038)
hsa-miRNA-1-3p	1.74	0.61	0.51	0.35	0.29	0.84
hsa-miRNA-10a-5p	1.01	0.64	0.57	0.63	0.56 (*p* = 0.018)	0.89
hsa-miRNA-92b-3p	1.13	0.88	0.72	0.77	0.64	0.82
hsa-miRNA-95-3p	1.18	2.61	0.93	2.21	0.79	0.36
hsa-miRNA-122-5p	1.09	1.65	1.07	1.52	0.99	0.65
hsa-miRNA-127-3p	1.31	0.79	1.30	0.60	0.99	1.65
hsa-miRNA-133a-3p	1.64	0.51	0.52	0.31	0.32	1.02
hsa-miRNA-133b	2.21	0.71	0.70	0.32	0.32	0.99
hsa-miRNA-136-5p	1.17	0.68	1.43	0.58	1.22	2.11
hsa-miRNA-154-5p	1.31	0.84	1.32	0.64	1.01	1.58
hsa-miRNA-186-5p	1.01	0.86	1.05	0.86	1.04	1.21 (*p* = 0.01)
hsa-miRNA-194-5p	1.12	2.47	2.55	2.21	2.28	1.03
hsa-miRNA-195-5p	1.00	1.25	1.46	1.24	1.45 (*p* = 0.02)	1.17
hsa-miRNA-365a-3p	1.07	0.87	0.67	0.82	0.62 (*p* = 0.02)	0.76
hsa-miRNA-382-5p	1.15	0.76	1.57	0.65	1.36	2.08 (*p* = 0.007)
hsa-miRNA-451a	1.10	2.01	1.29	1.83 (*p* = 0.029)	1.18	0.64
hsa-miRNA-500a-5p	1.09	0.77	0.65	0.71	0.59	0.84
hsa-miRNA-532-3p	1.03	0.96	0.74	0.93	0.71	0.77 (*p* = 0.038)
hsa-miRNA-574-3p	1.02	0.87	0.72	0.85	0.71 (*p* = 0.043)	0.83
hsa-miRNA-1974	1.39	3.20	1.73	2.31	1.25	0.54

MicroRNAs significantly (*p* < 0.05) or more than 1.5-fold up- or down-regulated comparing plasma samples from esophageal adenocarcinoma (EAC), Barrett’s esophagus (BE), and controls. Mean expression along with their fold induction are shown

Comparing plasma samples from EAC and controls, we identified one miRNA that was more than 1.5-fold up-regulated and seven miRNAs that were more than 1.5-fold down-regulated (Table 3). Particularly, miRNA-10a-5p and -365a-3p were significantly down-regulated in EAC compared to controls. An additional two miRNAs were significantly differentially expressed comparing the two groups.

Comparing plasma samples from EAC patients and BE patients, we found five miRNAs that were more than 1.5-fold up-regulated and four miRNAs that were more than 1.5 fold down-regulated (Table 3). Particularly, miRNA-382-5p and let-7e-5p were significantly and more than 1.5-fold up-regulated in EAC compared to BE. Additionally, two miRNAs were significantly differentially expressed comparing the two groups.

### Validation study of selected miRNAs as plasma marker candidates

We subsequently validated our miRNA profiling study results using plasma samples from another 41 BE and 59 EAC patients and 15 controls. Based on our miRNA profiling study, we selected six miRNAs that were used for the validation study: miRNA-95-3p, -133a-3p, -136-5p, -194-5p, -382-5p, and -451a. These miRNAs met the selection criteria: comparing the various groups were 1.5-fold induced/reduced and significantly differentially expressed or more than twofold induced/reduced in one or two of the groups (detailed selection criteria are described in Suppl. Fig. 2).

**Fig. 2:**
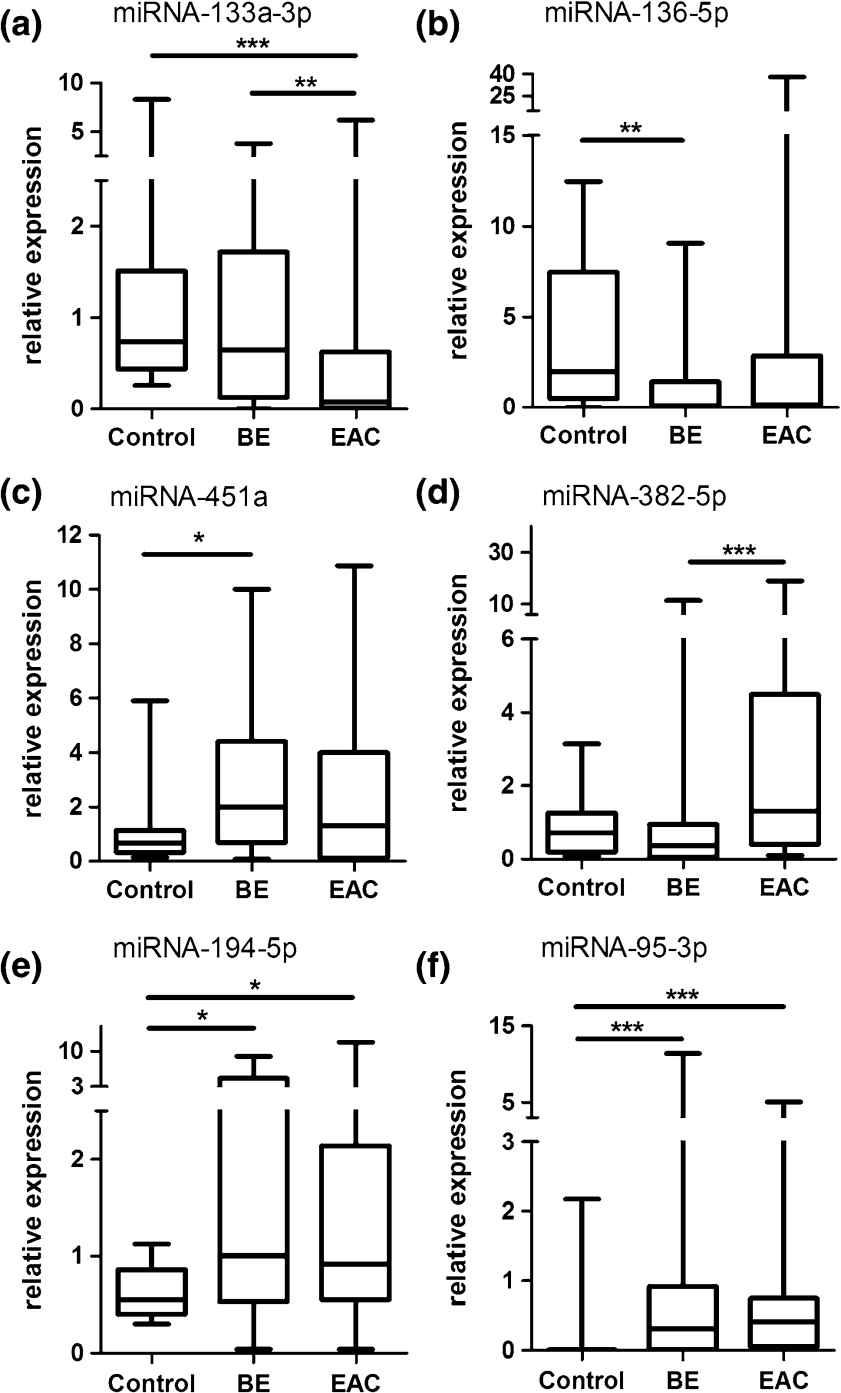
microRNA expression in plasma samples from patients with esophageal adenocarcinoma, Barrett’s esophagus, and normal controls; validation study. Validation by Q-RT-PCR of plasma samples from controls, *BE* Barrett’s esophagus and *EAC* esophageal adenocarcinoma patients. The upper and lower limits of the *boxes* and the *lines* inside the *boxes* indicate the 5th and 95th percentiles and the median, respectively. *p < 0.05, **p < 0.01, ***p < 0.001

Q-RT-PCR results indicated that miRNA-133a-3p was significantly higher expressed in plasma samples from controls (2.5-fold) and BE (2.1-fold) compared to EAC (Fig. 2a). miRNA-136-5p was significantly lower expressed in plasma samples from BE (threefold) compared to controls (Fig. 2b), whereas miRNA-451a was significantly higher expressed in BE (2.6-fold) compared to controls (Fig. 2c). miRNA-382-5p was significantly higher expressed in plasma samples from EAC compared to BE patients (2.4-fold) (Fig. 2d). In addition, miRNA-194-5p and -95-3p were significantly higher expressed in BE (4.2- and 4.4-fold, respectively) and EAC (3.2- and 2.8-fold, respectively), compared to controls (Fig. 2e, f). miRNA-136-5p showed an increased expression in EAC compared to BE (2.9-fold; Table 4). Known functional and expression data from these six miRNAs are summarized in Table 5.

**Table 4:** MicroRNA expression in plasma samples; validation study

MicroRNA ID	Mean expression controls	Mean expression BE	Mean expression EAC	Fold induction BE/controls	Fold induction EAC/controls	Fold induction EAC/BE
hsa-miRNA-95-3p	0.23	1.01	0.63	4.39 (*p* = 0.0009)	2.75 (*p* = 0.0002)	0.63
hsa-miRNA-133a-3p	1.33	1.11	0.53	0.83	0.40 (*p* = 0.0007)	0.48 (*p* = 0.0024)
hsa-miRNA-136-5p	3.94	1.31	3.80	0.33 (*p* = 0.0022)	0.96	2.91
hsa-miRNA-194-5p	0.62	2.58	2.01	4.17 (*p* = 0.0263)	3.25 (*p* = 0.0484)	0.78
hsa-miRNA-382-5p	0.93	1.39	3.34	1.50	3.60	2.41 (*p* = 0.0007)
hsa-miRNA-451a	1.09	2.84	2.36	2.61 (*p* = 0.0122)	2.17	0.83

Validation of miRNAs by Q-RT-PCR of plasma samples from controls, patients with Barrett’s esophagus (BE), and patients with esophageal adenocarcinoma (EAC). Mean expression along with their fold induction are shown

**Table 5:** Expression and function of miRNAs

MicroRNA	Presence in plasma/serum	Tissue expression/known function
hsa-miRNA-95-3p	_	Colorectal cancer: ↑ [49], oncogenic role [49]
hsa-miRNA-133a-3p	ESCC patients: ↓[22]	Ileal carcinoid tumor ↓[50], tumor-suppressive role [51]
hsa-miRNA-136-5p	_	Lung cancer: ↑[52], oncogenic role [52]
hsa-miRNA-194-5p	ESCC patients: ↑[23]	BE/EAC tissue: ↑[14]
hsa-miRNA-382-5p	Breast cancer patients: ↑[35]	Ovarian cancer: ↓[53], role in TGF-beta pathway [54]
hsa-miRNA-451a	Gastric cancer patients: ↑[36]	BE/EAC tissue: ↑[14]

*ESCC* esophageal squamous cell carcinoma, *BE* Barrett’s esophagus, *EAC* esophageal adenocarcinoma, *TGF*-*beta* transforming growth factor-beta

### Diagnostic potential of circulating miRNAs in plasma samples, validation study

**Fig. 3:**
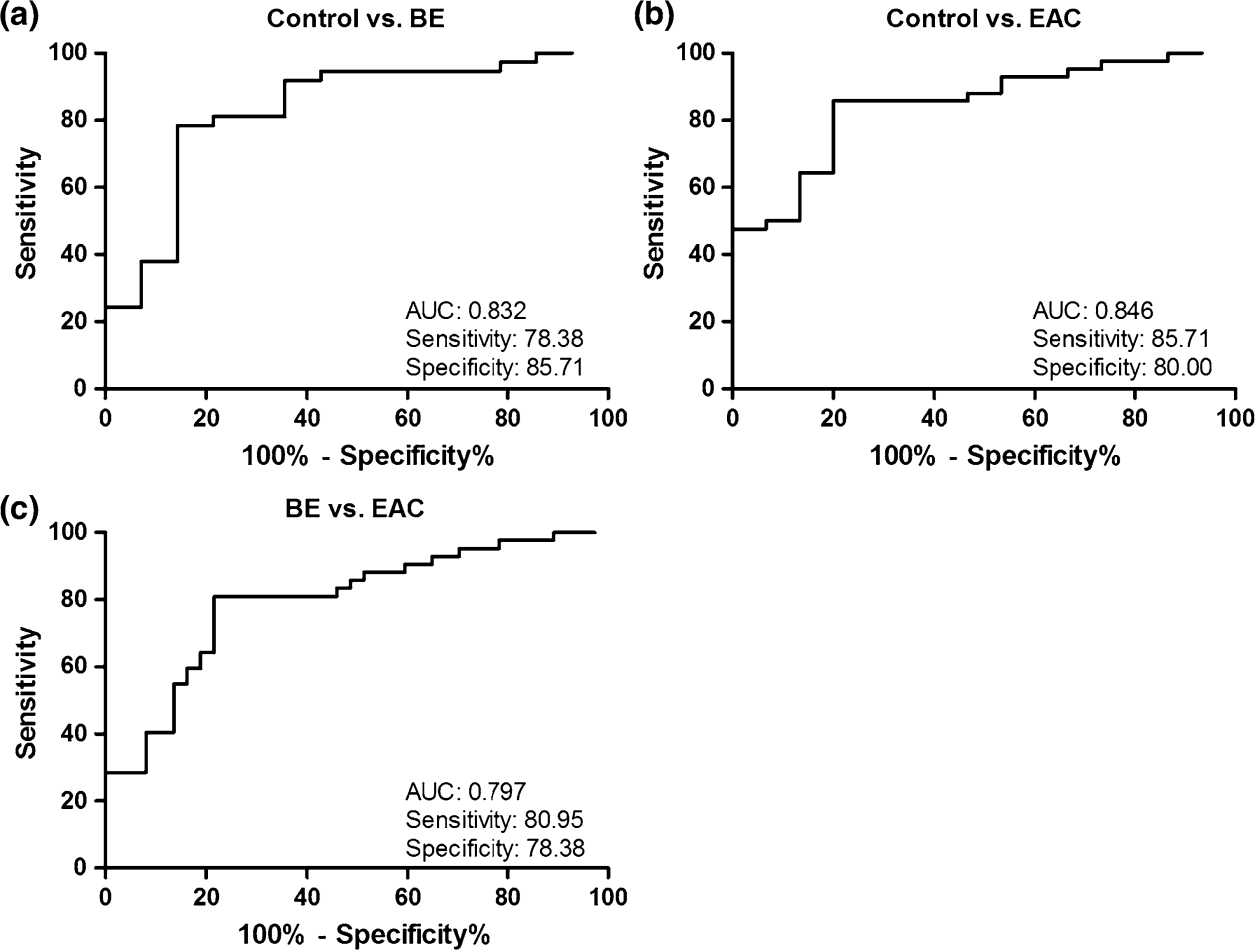
Receiver operating characteristic curve analysis for discriminating normal controls from patients with Barrett’s esophagus and esophageal adenocarcinoma. Evaluation of the diagnostic potential of microRNAs in the plasma samples from controls, *BE* (Barrett’s esophagus) and *EAC* (esophageal adenocarcinoma) patients. Presented *ROC* (receiver operating characteristic) curves show the greatest *AUC* (area under the curve) for distinguishing controls, BE, and EAC patients. All formulas used to combine the microRNAs were calculated by performing logistic regression analysis. **a** The ROC analysis to distinguish controls from BE patients. A combination of miRNA-95-3p, -136-5p, -194-5p, and -451a was used. The formula used: 0.391 + (0.734*miRNA-95-3p) + (−0.217*miRNA-136-5p) + (0.158*miRNA-194-5p) + (0.322*miRNA-451a). **b** Diagnostic performance of miRNA-133a-3p, -382-5p, and -451a for distinguishing controls from EAC patients. The formula used: −0.303 + (−0.703*miRNA-133a-3p) + (0.925*miRNA-382-5p) + (0.380*miRNA-451a). **c** ROC curve analysis showing the diagnostic performance of miRNA-133a-3p, -136-5p, -382-5p in order to distinguish BE from EAC patients. The formula used: 0.103 + (−0.557*miRNA-133a-3p) + (0.068*miRNA-136-5p) + (0.215*miRNA-382-5p)

To investigate the diagnostic potential of circulating miRNAs, we determined the discriminatory power between the various groups by performing logistic regression and ROC analysis using the Q-RT-PCR results from the validation study (Fig. 3). The most informative diagnostic panel to distinguish controls from BE was the combination of miRNA-95-3p, -136-5p, -194-5p, and -451a showing the highest AUC 0.832 (95 % CI 0.698–0.967) with a sensitivity of 78.4 % (95 % CI 61.8–90.2) and specificity of 85.7 % (95 % CI 57.2–98.2) (Fig. 3a). To distinguish EAC from controls the combination of miRNA-133a-3p, -382-5p, and -451a showed an AUC of 0.846 (95 % CI 0.738–0.954), a sensitivity of 85.7 % (95 % CI 71.5–94.6), and a specificity of 80.0 % (95 % CI 51.9–95.7) (Fig. 3b). The highest potential to discriminate between BE and EAC patients was found by using a combination of miRNA-133a-3p, -136-5p, and -382-5p, with an AUC of 0.797 (95 % CI 0.699–0.896), with a sensitivity of 81.0 % (95 % CI 65.9–91.4) and a specificity of 78.4 % (95 % CI 61.8–90.2) (Fig. 3c).

### Comparison of circulating and tissue miRNAs

Previously, our group performed miRNA expression profiling using esophageal squamous epithelium, BE, and EAC tissue biopsies [14]. We, therefore, compared the miRNA expression profiles of plasma samples described in the present study with the miRNA expression profiles of tissue samples described in the previous study [14]. We found that miRNA-194-5p and -451a were higher expressed in both tissue and plasma samples from BE compared to normal squamous esophagus tissue and plasma from controls. In addition, miRNA-194-5p was higher expressed in both tissue and plasma from EAC compared to normal squamous esophagus tissue and plasma from controls. miRNA-451a was found to be lower expressed in both tissue and plasma from EAC compared to BE tissue and plasma (Table 6).

**Table 6:** Comparison of miRNA profiles in tissue and plasma

	Tissue	Plasma
	BE/SQ	BE/controls
hsa-miRNA-194-5p	36.02	2.17
hsa-miRNA-451a	3.97	1.83
hsa-miRNA-133b	3.11	0.58
hsa-miRNA-1-3p	2.64	0.56
hsa-miRNA-10a-5p	2.24	0.55

	EAC/SQ	EAC/controls
hsa-miRNA-194-5p	18.62	2.21
hsa-miRNA-365a-3p	0.45	0.62
hsa-miRNA-133b	2.58	0.65
hsa-miRNA-1-3p	2.31	0.44

	EAC/BE	EAC/BE
hsa-miRNA-451a	0.48	0.61

MiRNAs more than twofold up- or down-regulated comparing tissue samples and miRNAs more than 1.5-fold up- or down-regulated comparing plasma samples from esophageal adenocarcinoma (EAC), Barrett’s esophagus (BE), and controls are shown

*BE* Barrett’s esophagus, *SQ* squamous epithelium, *EAC* esophageal adenocarcinoma

## Discussion

In this exploratory study we demonstrated that circulating miRNAs may have a promising diagnostic performance for the detection of BE and EAC. This raises the possibility of using such markers to develop a non-invasive and rapid diagnostic test for detecting BE and EAC. The investigation of plasma miRNAs as diagnostic markers is still in its early stages. However, considering the rapid advancements in this field, the development of non-invasive, blood-based miRNA diagnostics may well be successful in the near future. In comparison to the current routine surveillance endoscopy program with histopathological analysis of biopsies, a plasma test would by far be more convenient, less invasive, and possibly also cost-effective. In addition to plasma samples, serum samples can also be used and several studies have analyzed the expression of circulating miRNAs in plasma samples and compared them with serum samples and found little or no difference in circulating miRNA expression [18, 31].

BE is considered to be a complication of longstanding gastroesophageal reflux disease and is thought to develop from no dysplasia via low-grade dysplasia and high-grade dysplasia to EAC [32]. In the present study, we only included BE patients without dysplasia; however, future studies focusing on miRNA plasma levels in BE patients progressing from low-grade to high-grade dysplasia are required. Additionally we did not include patients with reflux disease, which is also a subgroup for which the determination of specific circulating miRNAs is of interest. Using circulating miRNAs for the detection of progression to EAC could be useful to detect EAC in patients already known with BE or as a general screening test for EAC. In addition, determining miRNA plasma profiles of all tumor stages separately could be of high interest. However, in our study, it was not possible to draw a conclusion related to tumor stages, since the number of patients in each group was not sufficient.

Several studies investigating circulating plasma miRNAs as potential markers to detect various types of cancer have shown promising results [20, 24, 33]. For example, a combination of two miRNAs (miRNA-21 and -375) has been suggested to have a predictive value for the detection of ESCC [20]. Several other miRNAs were found to be differentially expressed in plasma or serum of ESCC patients, compared to controls [20–23]. Two of the miRNAs in this study were also found in our dataset. First, miRNA-194-5p was found to be increased in ESCC patients [23], which is comparable to our results. Second, miRNA-133a-3p expression was found to be increased in ESCC [22], but we found miRNA-133-3p to be decreased in plasma from EAC. These two esophageal cancer types are fundamentally different in pathogenesis and tumor biology [34], which likely explains some of the differences in circulating miRNA expression. Of the other miRNAs in our dataset, miRNA-382-5p was also found to be increased in plasma of breast cancer patients and included in a panel of miRNAs to detect breast cancer [35]. miRNA-451a was elevated in plasma of gastric cancer patients and proposed to be part of a panel to diagnose early stage gastric cancer (Table 5) [36].

In Table 6, we compared miRNA expression in plasma and tissue from controls and patients with BE and EAC. miRNA-194-5p, -451a, and -365a-3p expression showed a similar expression pattern in tissue and plasma. Other miRNAs showed contradictory results, with an increase in BE or EAC tissue and a concomitant decrease in BE or EAC plasma. Since the exact origin and function of circulating miRNAs is unknown [37, 38], we are unable to explain the difference in expression.

MiRNA measurement in plasma is affected by the occurrence of hemolysis or the presence of residual platelets in the sample [39], because red blood cells and platelets are known to contain miRNAs [40, 41]. In order to remove residual platelets we performed post-storage centrifugation of the plasma samples. Hemolysis of whole blood samples results in an increase of erythrocyte-specific miRNAs (miRNA-16-5p and miRNA-144-3p), which are not disease-specific [42, 43]. In the current study we identified miRNA-451a as one of the markers. We realized that the results on miRNA-451a should be interpreted with caution, since hemolysis can greatly affect the presence of this miRNA [42]. To determine whether the differential miRNA-451a expression was due to hemolysis, we measured the presence of miRNA-16-5p. Our validation samples did not show an effect on miRNA-16-5p expression (data not shown) and, therefore, we concluded that our samples were free of hemolysis.

Although our results are promising, we are aware of some limitations. First, we included significantly more females in the control group. From a Chinese study it is known that 90 of 101 miRNAs are present in both male and female serum, but miRNA-100, -184, and -923 are male-specific, while miRNA-222 is female-specific [44]. These miRNAs were not induced or reduced in our groups; therefore, we concluded that gender probably did not have an effect on our results. In addition, patients in the control group were younger than the patients in the BE and the EAC groups. This is a result of our inclusion criteria, in which controls were excluded when they had reflux symptoms. Previously, Noren Hooten et al. found five miRNAs in plasma to be differently present in serum of young and old people, using next generation sequencing [45]. We did not observe differential expression of these miRNAs comparing the various groups in our study. It was challenging to include persons with similar characteristics as the controls, particularly because both BE [46] and reflux symptoms [47] are more common among older males. We divided normal controls, BE, and EAC patients in various age groups, males, and females; miRNA expression results are depicted in Supplemental Figs. 5–10. Thirdly, in the profiling study, we only chose to test 175 miRNAs that were known to be present in human plasma samples. The miRNA research field is developing fast, and new miRNAs are still (to be) identified. The number of known miRNAs has increased dramatically over the last few years. In order to discover all circulating miRNAs differentially expressed, high-throughput sequencing should be used in the future. In addition, in large-scale profiling studies, it is useful to use a false discovery rate approach because multiple testing can lead to false discoveries [48]. Here we chose to describe the actual *p* value (Table 3). Finally, literature proving reproducibility of circulating miRNAs is lacking. Therefore it is important to analyze if circulating miRNAs are stably expressed over time without disease progression.

Although the number of studies published on circulating miRNAs as potential markers for cancer is increasing, data normalization remains an important issue. In tissue miRNA analysis, U6snRNA is commonly used for normalization; however, its expression is not stable in plasma [44]. There are a few approaches that can be used to minimize experimental variation, for example, spike-in RNAs [18]. During our quality control experiments, we included an artificial spike-in RNA and achieved high technical quality. In addition, we used NormFinder to identify the best normalizer [29]. This showed that the average of assays detected in all samples could be used for normalization in our miRNA expression profiling dataset and that a set of five miRNAs (miRNA-93-5p, -103a-3p, -106a-5p, -423-3p, and -423-5p) could be used for validation [30]. These miRNAs were stably expressed in each group during our initial miRNA expression profiling (Suppl. Fig. 3). However, in the future, a consensus endogenous control suitable for normalization needs to be established.

In summary, we performed a profiling study to identify circulating miRNAs that are able to distinguish BE and EAC patients from controls and validated this in a larger cohort. In the future, this may have important clinical implications as it may be able to develop minimally invasive markers for diagnosing BE and EAC. These markers could make future screening and surveillance protocols that are based on upper endoscopy more (cost-)effective assigning patients to endoscopy when circulating miRNAs suggest that they are at risk of having BE and/or developing EAC, whereas in those not at risk, endoscopy is not indicated. Nevertheless, for now, further validation in larger and independent cohorts is required.

## Electronic supplementary material

Below is the link to the electronic supplementary material.

Supplementary material 1 (DOCX 845 kb)
