# Supplementary material for: Profiling of circulating microRNAs in patients with Barrett’s esophagus and esophageal adenocarcinoma
Source: J Gastroenterol. 2015 Nov 19;51:560–70. doi: 10.1007/s00535-015-1133-5 (PMC4880635; doi:10.1007/s00535-015-1133-5)
Supplement: Supplementary file 1 — Supplementary material 1 (DOCX 845 kb) [file 535_2015_1133_MOESM1_ESM.docx]

**SUPPLEMENTARY FIGURES**

**Supplementary figure 1: Study design**

**
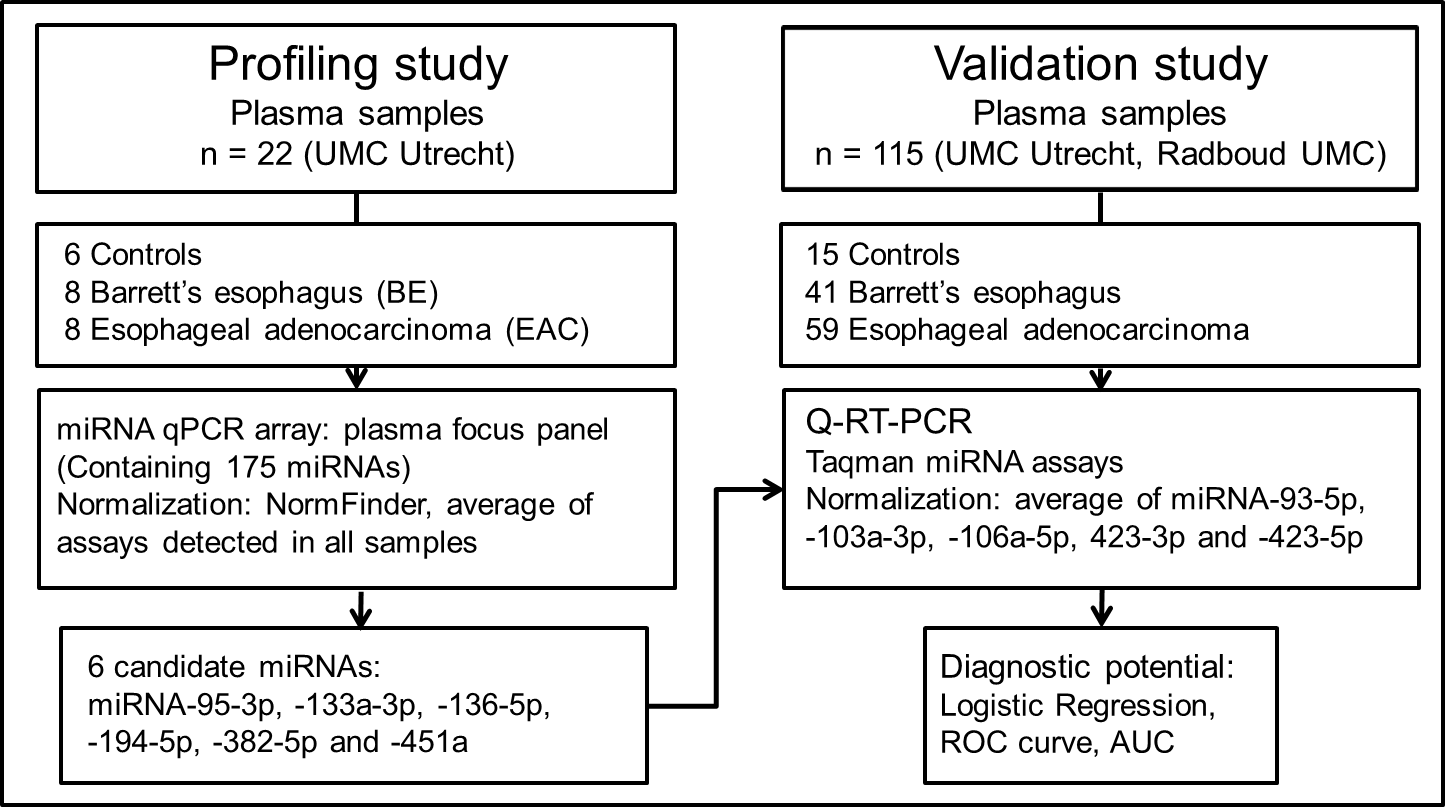
**

Legend: We divided the study into a profiling and validation study.

**Supplementary figure 2: Selection criteria for the miRNAs in the validation study**


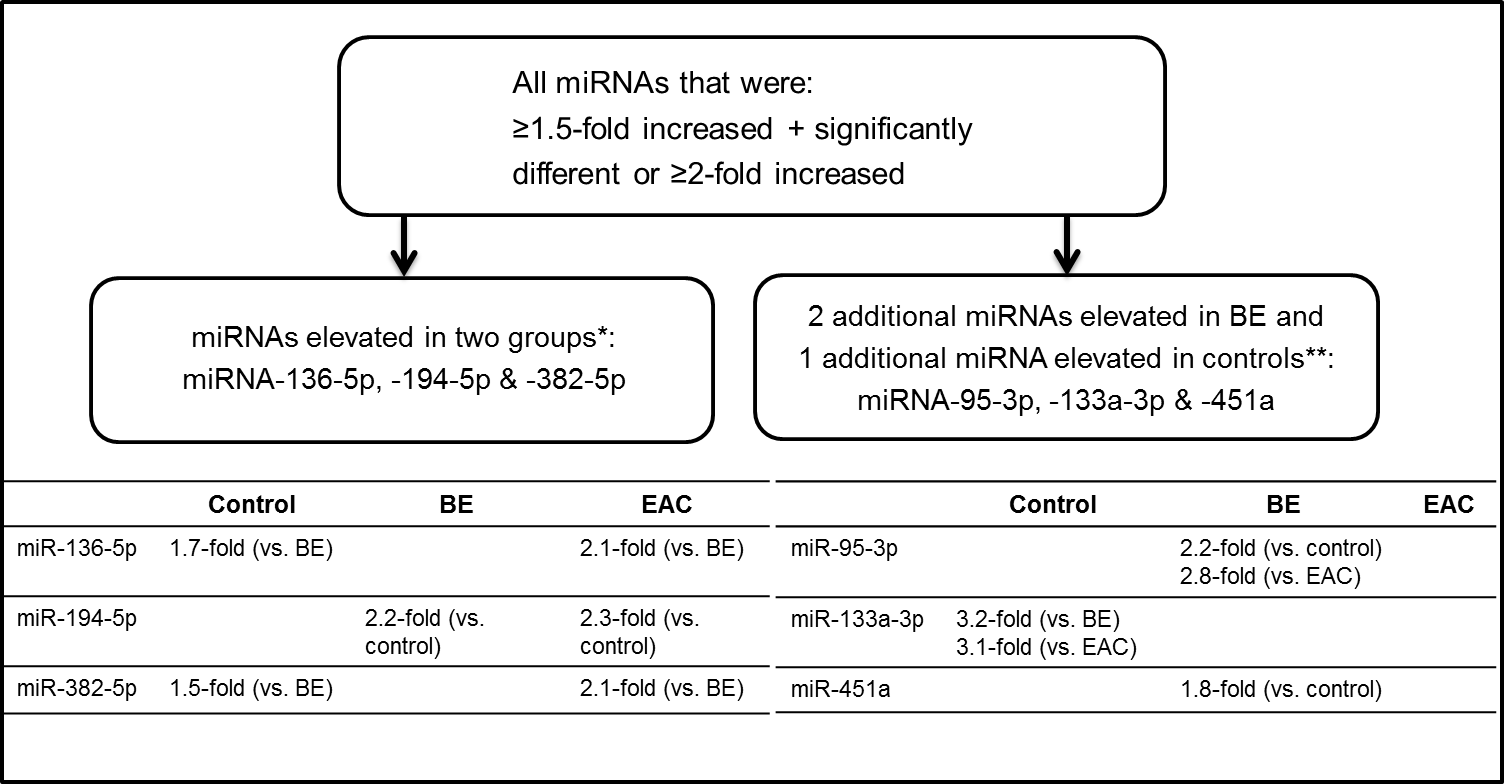


Legend: This figure shows the selection criteria for the 6 miRNAs used for validation. After the selection of all miRNAs that were elevated in two groups (*; miRNA-136-5p, -194-5p and -382-5p), we selected 2 additional miRNAs elevated in Barrett’s esophagus (BE; miRNA-95-3p and -451a) and 1 additional miRNA elevated in controls (miRNA-133a-3p) (**). miRNA-136-5p, -194-5p and -382-5p were all elevated in esophageal adenocarcinoma (EAC). Additionally, two of these miRNAs (miRNA-136-5p and -382-5p) were elevated in controls versus BE, whereas miRNA-194-5p was elevated in BE versus controls. Therefore, we selected 1 additional miRNA elevated in controls (miRNA-133a-3p) and two additional miRNAs that were elevated in BE (miRNA-95-3p and -451a). These additional miRNAs were chosen, because they fitted the selection criteria best. miRNA-133a-3p was chosen because this miRNA showed the highest increase in controls compared to both BE and EAC. miRNA-451a was chosen since this miRNA has been found to be increased in BE tissue.[[14](#_ENREF_14)]

**Supplementary figure 3: Evaluation of microRNA expression, used for normalization purposes, in plasma samples from patients with esophageal adenocarcinoma, Barrett’s esophagus and controls; profiling study**

**
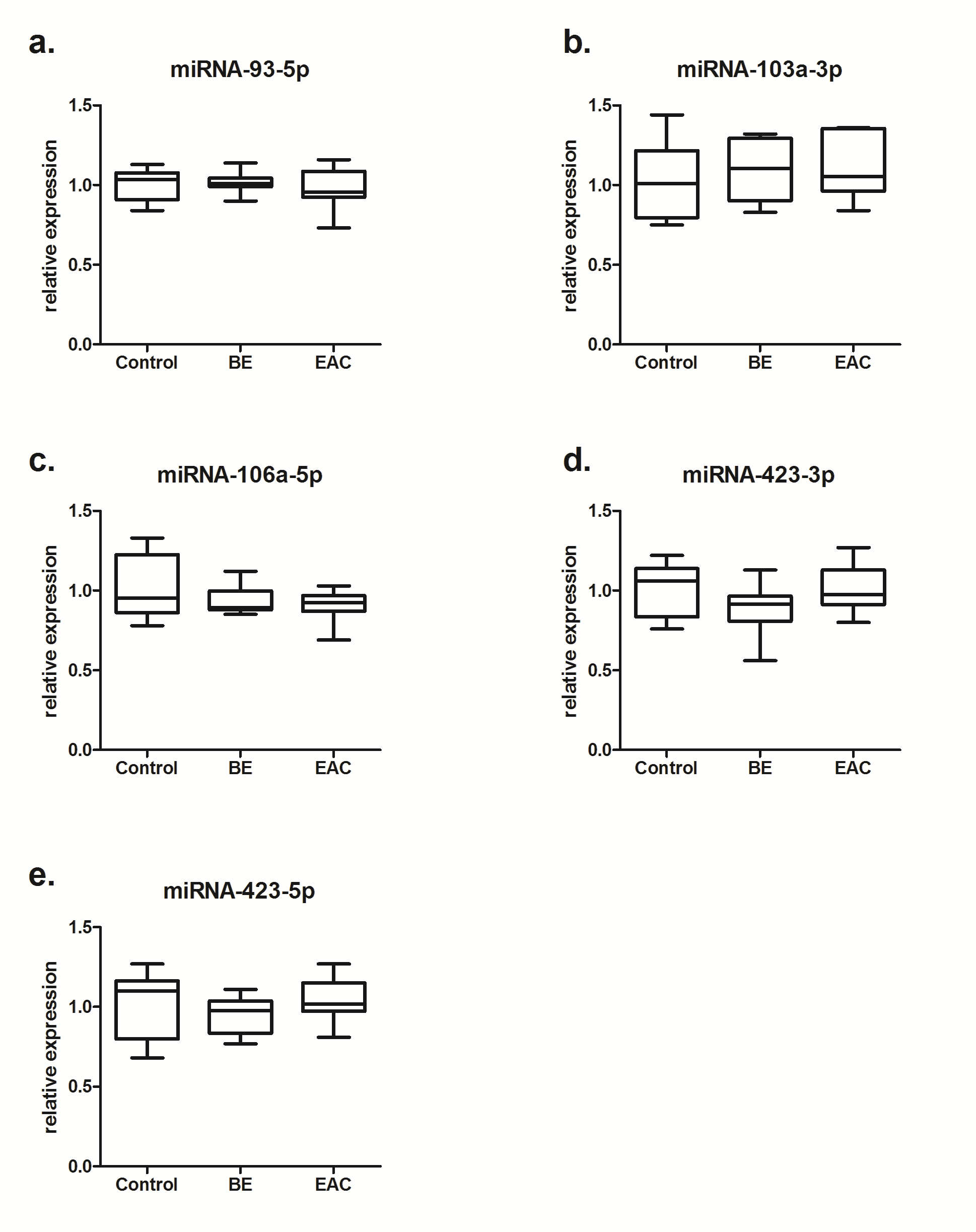
**

Legend: miRNA expression in plasma samples from controls, Barrett’s esophagus (BE) and esophageal adenocarcinoma (EAC) patients in the profiling study. Depicted are the miRNAs chosen for normalization in the validation study. The upper and lower limits of the boxes and the lines inside the boxes indicate the 5^th^ and 95^th^ percentiles and the median, respectively.

**Supplementary figure 4: Quality control of samples used for plasma miRNA profiling**

**
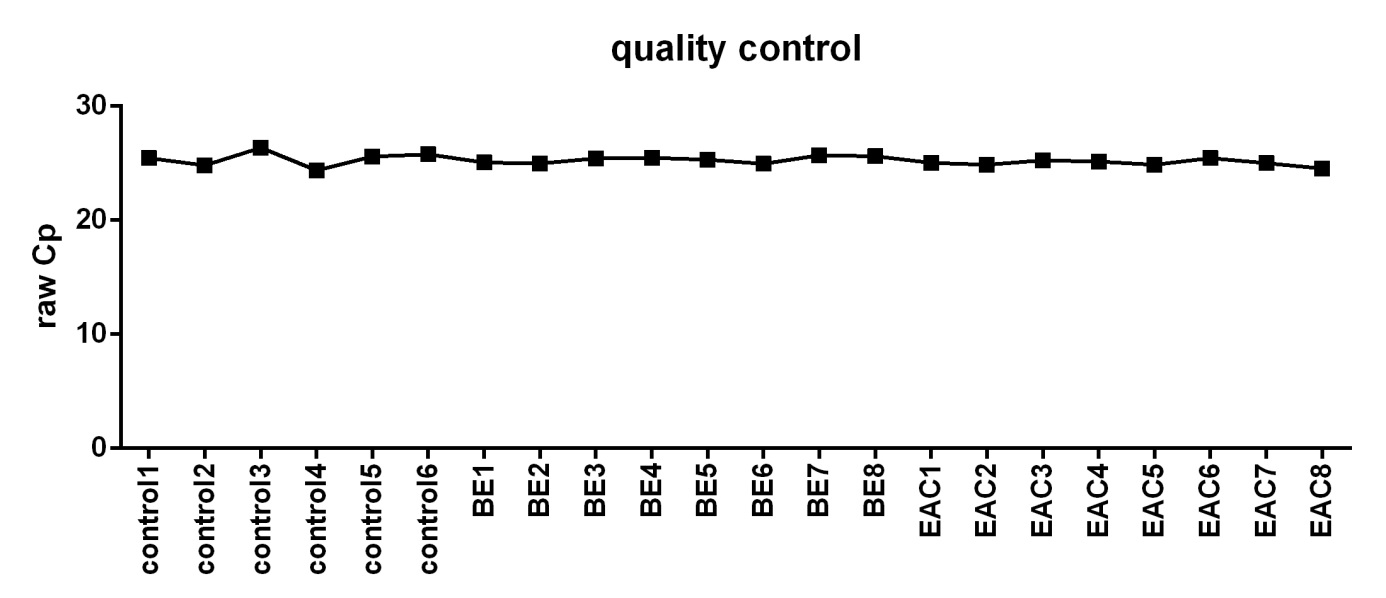
**

Legend: RNA quality of all samples used for miRNA profiling study. This figure shows the raw Cp values of the synthetic spike-in RNA (Sp6).

**Figure 5: MicroRNA expression in plasma samples divided in age groups.**



Legend: Circulating microRNA expression from normal controls divided in various age groups (19-39, 40-49, 50-59, 60-69 and 70-81). The upper and lower limits of the boxes and the lines inside the boxes indicate the 5^th^ and 95^th^ percentiles and the median, respectively. N is the number of controls or patients in each group.

**Figure 6: MicroRNA expression in plasma samples divided in age groups.**





Legend: Circulating microRNA expression from Barrett’s esophagus patients divided in various age groups (19-39, 40-49, 50-59, 60-69 and 70-81). The upper and lower limits of the boxes and the lines inside the boxes indicate the 5^th^ and 95^th^ percentiles and the median, respectively. N is the number of controls or patients in each group.

**Figure 7: MicroRNA expression in plasma samples divided in age groups.**





Legend: Circulating microRNA expression from esophageal adenocarcinoma patients divided in various age groups (19-39, 40-49, 50-59, 60-69 and 70-81). The upper and lower limits of the boxes and the lines inside the boxes indicate the 5^th^ and 95^th^ percentiles and the median, respectively. N is the number of controls or patients in each group.

**Figure 8: MicroRNA expression in plasma samples from males and females.**

**

**

Legend: Circulating microRNA expression from normal controls divided in males and females. The upper and lower limits of the boxes and the lines inside the boxes indicate the 5^th^ and 95^th^ percentiles and the median, respectively. N is the number of controls or patients in each group.

**Figure 9: MicroRNA expression in plasma samples from males and females.**

**

**

Legend: Circulating microRNA expression from Barrett’s esophagus patients divided in males and females. The upper and lower limits of the boxes and the lines inside the boxes indicate the 5^th^ and 95^th^ percentiles and the median, respectively. N is the number of controls or patients in each group.

**Figure 10: MicroRNA expression in plasma samples from males and females.**

**

**

Legend: Circulating microRNA expression from esophageal adenocarcinoma patients divided in males and females. The upper and lower limits of the boxes and the lines inside the boxes indicate the 5^th^ and 95^th^ percentiles and the median, respectively. N is the number of controls or patients in each group.
